# Knee Extensor Apparatus Reconstruction with Allograft after Patellar Resection: A Case Report

**DOI:** 10.15388/Amed.2024.31.1.24

**Published:** 2024-02-27

**Authors:** Fabio Cosseddu, Martina Cordoni, Elena Bechini, Edoardo Ipponi, Francesco Rosario Campo, Antonio D’Arienzo, Lorenzo Andreani

**Affiliations:** 1 Department of Orthopedics and Trauma Surgery, University of Pisa, Pisa, Italy

**Keywords:** Extensor Apparatus, Patella, Bone Metastasis, Allograft, Rehabilitation, tiesiamasis aparatas, blauzdikaulis, kaulų metastazės, alograftas, reabilitacija

## Abstract

**Background:**

The extensor apparatus of the knee can be thought of a chain that transmits the muscular strength developed by the quadriceps muscles to the proximal tibia. This complex is essential to allow the extension of the tibia over the femur, being essential to provide knee mobility and stability. In case of lesions which irreparably damage the patella, such as a locally aggressive bone tumor, it is necessary to restore both the apparatus’ anatomical continuity and its strength.

**Case report:**

A 39-years-old Caucasian man with a history of lung carcinoma developed atraumatic swelling and soreness in his left knee. Imaging evidence reported a degeneration of the left patella. We performed an en bloc resection of the patella and the nearby soft tissues of the extensor apparatus. The resulting gap was fulfilled with a massive allograft consisting of a quadriceps tendon, a patella and a patellar ligament. No complication or local recurrences were observed. At the patient’s latest follow-up, he did not have any extension lag and quadriceps strength was completely restored.

**Conclusion:**

Massive allografts can represent a reliable alternative for the reconstruction of the patella and the knee extensor apparatus in orthopedic oncology.

## Abstract (it)

**Įvadas:**

Kelio tiesiamąjį aparatą galima teigti esant grandine, kuri perduoda keturgalvio raumens raumenų sukurtą jėgą proksimaliniam blauzdikauliui. Šis kompleksas yra labai svarbus, kad blauzdikaulis galėtų išsitempti virš šlaunikaulio, ir yra būtinas kelio judrumui ir stabilumui užtikrinti. Esant pažeidimų, kurie negrįžtamai pažeidžia girnelę, pavyzdžiui, vietinio agresyvaus kaulų naviko atveju, būtina atkurti anatominį kelio tiesiamojo aparato vientisumą ir jo tvirtumą.

**Atvejo aprašymas:**

39 metų baltaodžiui, kuriam anksčiau buvo diagnozuota plaučių karcinoma, atsirado kairės kojos kelio atraminis patinimas ir skausmingumas. Vaizdiniais duomenimis nustatėme kairiojo kelio girnelės degeneraciją. Atlikome *en bloc* kelio girnelės ir šalia esančio tiesiamojo aparato minkštųjų audinių rezekciją. Atsiradusi spraga buvo užpildyta masyviu alografiniu transplantatu, kurį sudarė keturgalvio raumens sausgyslė, girnelė ir girnelės raištis. Komplikacijų ar vietinių recidyvų nebuvo. Paskutinio stebėjimo metu pacientas neturėjo jokio tiesimo sutrikimo, o keturgalvio raumens jėga buvo visiškai atkurta.

**Išvada:**

Masyvūs alograftai gali būti patikima alternatyva kelio girnelės ir kelio girnelės tiesiamojo aparato rekonstrukcijai ortopedinėje onkologijoje.

## Introduction

The extensor apparatus of the knee, consisting of the quadriceps tendon, the patella, and the patellar tendon, connects the quadriceps muscle to the tibial tuberosity. It allows the extension of the tibia over the femur and stabilizes the whole knee articulation by transmitting the muscular strength developed by the quadriceps muscle to the tibia. Therefore, neoplastic lesions arising from the quadriceps or patellar tendon, as well as bone tumors of the patella, can lead to functional impairment of the knee, with an extension deficit and an inability to maintain an upright position [1]. These lesions often require massive resections to achieve wide margins, and a proper anatomical reconstruction is fundamental to restoring the continuity and function of the extensor apparatus [2]. Despite the recent advances in surgical techniques, bioengineering, and bone allograft banking, the reconstruction of the extensor apparatus after wide resection still represents one of the most challenging procedures in orthopedic oncology [2, 3]. To this date, a consensus about the best reconstructive approach still needs to be established. We report a case of metastatic lung carcinoma localized in the left patella treated with resection of the knee extensor apparatus and reconstruction with allograft.

## Case presentation

This report has been performed following the ethical standards laid down in the 1964 Declaration of Helsinki and its later amendments.

The patient consented to the treatment and the article’s drafting.

Our patient is a 39-year-old male with a history of adenoid cystic carcinoma of the left lung, successfully treated with pneumonectomy and postoperative radiotherapy. He came to our attention due to persistent left knee pain; the soreness had been increasing in frequency and magnitude during the previous months, being present during both day and night hours and limiting the patient’s daily activities. Symptoms were associated with a painful palpable swelling on the surface of the left patella. The left knee’s range of motion (ROM) was incomplete, with pain at maximum degrees of flexion and extension. The preoperative Musculoskeletal Tumor Society Functional Score was 11 out of 30.

**Figure 1:**
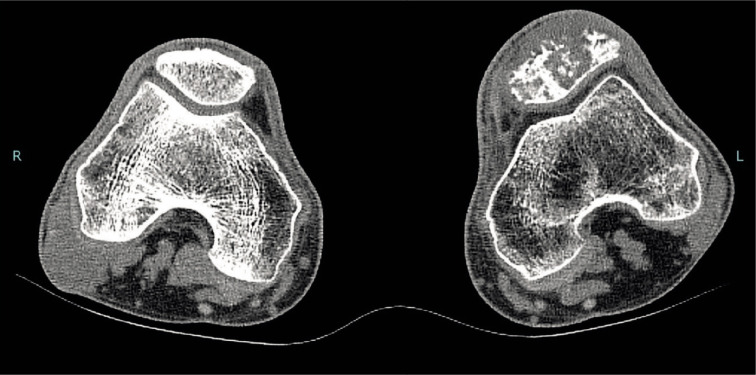
A CT scan that shows a degeneration of the left patella.

The patient had already undergone a CT scan and MRI evaluation, which highlighted the presence of a 5 cm wide osteolytic lesion associated with focal degeneration of the left patella (Figure 1).

Patella cortical bone showed significant erosion, with several areas of discontinuity, attributable to disease local growth, which did not allow for a curettage. Considering imaging evidence, the patient’s previous pathological history, and general clinical conditions, we opted for wide patella resection and subsequent replacement with an allograft. Therefore, we ordered from our local bone bank an allograft consisting of a quadriceps tendon, patellar ligament, and a patella comparable to the contralateral one in size and shape.

**Figure 2:**
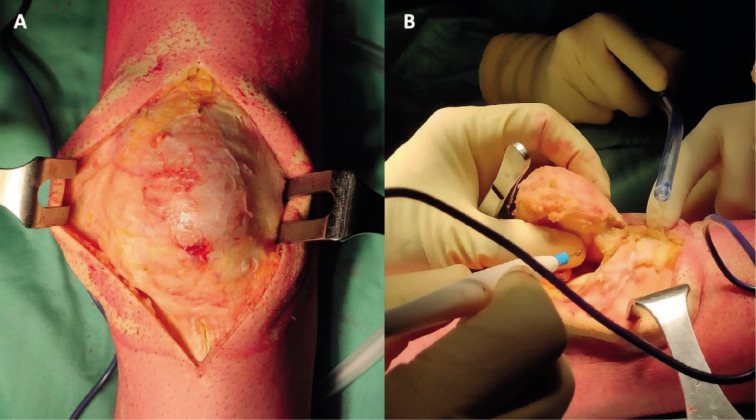
Surgical access with extensive exposition of the patella, which appears enlarged and deformed (A). The access was used to isolate the degenerated patella from the surrounding soft tissues (B).

***Surgical technique*.** The procedure was performed with the patient lying supine on the surgical table. A pneumatic tourniquet was set at the left limb root and activated before the skin incision. A direct longitudinal approach to the anterior knee centered on the patella was performed: the skin incision was extended for about 25 cm to allow complete exposure of the patella and its surrounding tissues (Figure 2A). The patella was then isolated from the surrounding soft tissues while preserving the quadriceps tendon and patellar ligament (Figure 2B).

**Figure 3:**
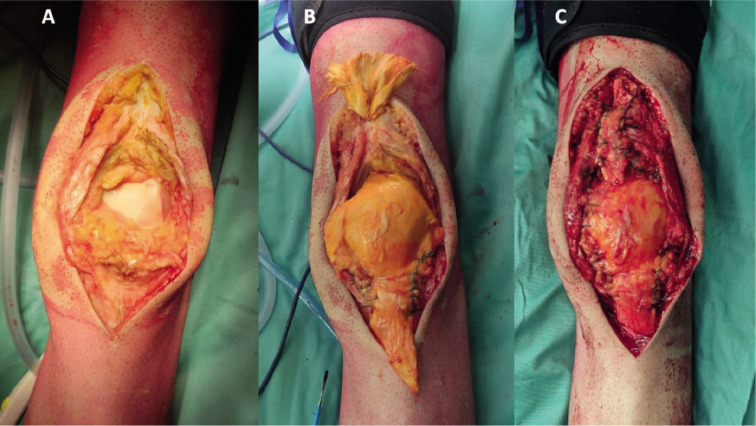
Pictures showing the anterior knee after the resection (A), After one the allograft had been sutured in its distal end and tunneled within the native quadriceps tendon (B) and once it had been completely sutured to restore the continuity of the extensor apparatus (C).

The removed patella (Figure 3A) was then delivered to our Pathological Anatomy division, which confirmed the presumptive diagnosis of skeletal localization of adenoid cystic carcinoma. The remaining patellar ligament was split, and the allograft was set in place of the removed patella. Distally, the patellar ligament of the graft was set between the native ligament’s medial and lateral segments and sutured with fiber wires (Figure 3B). Proximally we made a hole in the native quadriceps tendon, through which we passed the rolled proximal segment of the allograft quadriceps tendon (Figure 3C). Local blood loss was prevented with careful hemostasis of the surgical bed. The wound was closed with an intradermal suture.

Finally, we tested allograft resistance through passive range of movement. Patient’s knee showed complete passive flexion-extension mobility and was stable to varus and valgus stresses.

In order to protect the new extensor apparatus, the knee was immobilized in a long articulated knee brace that was maintained at 0° of flexion for 30 days. Weight-bearing was avoided for the first 30 days after surgery.

The brace was kept in place for the following two months and a half, with progressive increments of the allowed range of motion (ROM) and progressive weight bearing, as shown in Table 1.

**Table 1:** A resume of our patient’s rehabilitation protocol.

DAYS	LOAD	KNEE R.O.M.
0–30	None*	None*
30–37	15 kg	0–30°
38–44	30kg	0–45°
45–52	45kg	0–60°
53–59	50kg	0–90°
60–90	50kg	0–120°
90 +	50kg**	0–120°**

* Within an articulated knee brace ** Without braces

Within three months after surgery, the patient abandoned crutches and was allowed to walk autonomously.

At latest follow-up, twelve months after surgery, patient’s knee had an active painless range of motion of 0–120°, extension strength was restored (MRC 5/5), and his left limb could successfully support his total weight of 50 Kg. Flexion and extension strength were comparable to the contralateral knee. No major local or systemic complications occurred postoperatively. No local recurrence was diagnosed through the patient’s postoperative intercourse. The patient showed satisfaction with the functional results of our treatment which allowed him to return to his daily activities.

## Discussion

The reconstruction of the extensor apparatus in the knee is challenging even for the most experienced orthopedic surgeon, as the necessity to achieve wide resection margins often translates into massive gaps in the extensor apparatus of the knee. Although the resection of the patella without subsequent reconstructions has been described in literature [1, 2], in case of extensive tissue gaps surgeons are generally called to replace the native extensor apparatus with substitutes that could restore the anatomical continuity and the functionality [1, 2]. The augments of choice should be robust enough to withstand the repetitive strain and loads of the knee. To this date, a consensus treatment for extensor apparatus reconstruction has yet to be established. Some case reports and technical notes so far published regard lesions confined to the anterior knee or associated with distal femur or proximal tibia that required megaprosthetic reconstructions. Several solutions have been proposed and described through the last decades, including systemic and prosthetic augments [3, 4], autologous tissues [5], and allografts [4, 6-8]. Reconstructive solutions include biological [3, 4, 6-8] and nonbiological implants [4, 5], both of them with their pros and cons. Although synthetic augments often have good mechanical properties, they lack bio-integrative properties, and their performances may decrease over the years. Biological reconstructions have a bio-integrative potential to bridge the augment with the receiving site, leading to a better functional horizon in the mid- and long-term scenario.

Selected allografts harvested from the same body segment represent suitable reconstructive options due to their length, mechanical strength, and bio-integrative potential. Although expensive and burdened by the risk of complications such as infections, nonunions, ruptures, or fractures, tendon and bone allografts have some advantages in reconstructing large anatomical segments. Compared to autografts, allografts avoid sacrificing a donor site, minimizing the risk of complications and reducing surgical times. Furthermore, allogeneic augments can be chosen in size and features in order to respect the patient’s anatomy, even when massive resection is required to achieve wide margins and eradicate the neoplasm [9]. Finally, from a biological point of view, allografts provide a good bio-integrative potential once placed in the receiving site. Despite the absence of proper internal vascularization, allograft surfaces are biological scaffolds that will eventually be inhabited by receiving patient cells. The process consists of three biological stages: an early and acute inflammatory phase with ischemic necrosis, a phase of cell recruitment and chronic inflammation with revascularization and collagen remodeling, and finally a ligamentization phase. The result is an anatomical and functional bridging between the native tissues and the graft [9].

Although modern literature has evidence of cases treated with grafts that did not include a patella [4,6], this latter one has a strategic role during the knee extension, working as a pulley to maximize the effectiveness of quadriceps’ contractile strength [10].

The first case with a malignant bone tumor of the patella and the extensor apparatus treated with massive resection and a massive allograft that included a patella was reported in 2009 by Cho et al. [6]. Three years after surgery, the authors obtained good functional results with a graft consisting of a quadriceps tendon, a patella with a patellar tendon, and the medial and lateral retinacula. In 2019, Gómez-Palomo et al. [8] performed a similar reconstruction for a 24 year-old case with a patellar osteosarcoma. Within five years after surgery, their patient highlighted good functional and clinical outcomes. Although our and theirs cases have the same treatment rationale and share the same surgical approach, our surgical technique was slightly different. The allograft used by Gómez-Palomo et al. had a relatively short-sized quadriceps tendon, while ours was long enough to tunnel through what remained of the native quadriceps tendon. The distal anchorage was also different. Their graft had a complete patellar ligament and a fragment of tibial tuberosity, which were anchored to the native tibia with two screws. In our case, the native patellar ligament was largely preserved, allowing us to split and suture it to the allograft’s patellar ligament. Despite these variations, the clinical results were similar and encouraging, as our patient could return to his daily living activities and was satisfied with the results. The good outcomes of our patient, in terms of both strength and range of motion, support the effectiveness of allograft reconstructions of the extensor apparatus also for cases with metastatic bone tumors.

The mechanical and biological properties of the allograft, alongside a careful and personalized rehabilitation protocol focused on progressive articular mobilization and weight bearing, led to good mid-term functional outcomes.

## Conclusion

Several reconstructive solutions to replace the patella and the extensor apparatus of the knee have been described in modern literature. Our outcomes suggest that an allograft composed of a quadriceps tendon, patella, and patellar ligament can represent a suitable reconstructive option for patients undergoing wide total patellectomy due to malignant and locally aggressive bone tumor.
